# Stemness genes and miR-1247-3p expression associate with clinicopathological parameters and prognosis in lung adenocarcinoma

**DOI:** 10.1371/journal.pone.0294171

**Published:** 2023-11-10

**Authors:** Shiwani Limbu, Kara E. McCloskey

**Affiliations:** 1 Quantitative and System Biology Program, University of California, Merced, Merced, CA, United States of America; 2 Materials Science and Engineering Department, University of California, Merced, Merced, CA, United States of America; Baylor College of Medicine, UNITED STATES

## Abstract

Lung cancer makes up one-fourth of all cancer-related mortality with the highest mortality rate among all cancers. Despite recent scientific advancements in cancer therapeutics, the 5-year survival rate of lung adenocarcinoma (LUAD) cancer patients remains below 15 percent. It has been suggested that the high mortality rate of LUAD is linked to the acquisition of progenitor-like cells with stem-like characteristics that assist the whole tumor in regulating immune cell infiltration. To examine this hypothesis further, this study mined several databases to explore the presence of stemness-related genes and miRNAs in LUAD cancers. We examine their association with immune and accessory cell infiltration rates and patient survival. We found 3 stem cell-related genes, ORC1L, KIF20A, and DLGAP5, present in LUAD that also correlate with changes in immune infiltration rates and reduced patient survival rates. Additionally, the modulation in myeloid-derived suppressor cell (MDSC) infiltration and miRNA hsa-mir-1247-3p mediated targeting of tumor suppressor SLC24A4 and oncogenes RAB3B and HJURP appears to primarily regulate LUAD patient survival. Given these findings, hsa-mir-1247-3p and/or its associated gene targets may offer a promising avenue to enhance patient survivability.

## Introduction

Lung cancer corresponds to 25% of all cancer deaths [1], the greatest mortality rate among all cancers [2]. The two most common types of lung cancer are lung adenocarcinoma (LUAD) and lung squamous cell carcinoma (LUSC) [3], with LUAD recently replacing squamous cell cancer as the most prevalent non-small cell cancer in the United States. Despite recent scientific advancements in the development of anti-cancer therapeutics, the 5-year survival rate of LUAD cancer patients remains less than 12% to 15% [4].

One hypothesis for the high mortality rate of LUAD is its ability to evade the body’s immune system [5]. Cancer progression is marked by the de-differentiation of tissue-specific cells and acquisition of progenitor-like cells with stem-like characteristics. These cancer stem cells (CSC) [6] are then able to assist the whole tumor [7] with proliferation, progression and propagation [8]. Moreover, stem cells and CSCs in cancers correlate with the rate and type of immune responses [9], tumor metastasis [10], multi-drug resistance [11], and low cancer patient survival [12, 13]. The relationship between CSCs, stem cell-related genes, and cancer therapeutics has been widely investigated in a number of cancers with respect to potential biomarkers [14, 15], the tumor microenvironment (TME) [15, 16], and immune cell infiltration [17, 18]. CSCs have also been shown to be resistant to traditional therapies, correlating with enhanced epithelial to mesenchymal (EMT) properties, reduced DNA repair mechanisms, enhanced expression of ATP-binding cassette (ABC) membrane transporters, activation of several survival signaling pathways, and increased immune evasion [19].

Because immune cell infiltration affects cancer progression [20–22], immune cells have been proposed as possible delivery vehicles for gene therapy [23]. HSCs can infiltrate tumors to fight disease progression, but exhibit only limited infiltration in lung tumors [24]. Conversely, immune cells that enable cancer progression include MDSCs that can suppress T cell activation, induce other immune-suppressive cell populations, regulate inflammation, and promote the switching of the immune system to tolerate tumor growth [25, 26], and Th2 T cell CD4+ (Th2) cells promotes angiogenesis and inhibit cytotoxic T-cell immune responses [27]. Th2 cells regulate humoral immune response and produce interleukin 4 and 10 [28], which in turn reduces the normal Th1 cytotoxic activity [28] that can kill cancer cells [29]. Moreover, a negative relationship between cancer patient survival probability and Th2 infiltration rates has been demonstrated in LUAD patients [22, 30].

In addition to immune cell infiltration, the production of inflammatory molecules and other changes in gene expression and microRNAs (miRNAs) can act as a key cell signaling molecules in cancer initiation and metastasis [31]. miRNAs can change gene expression by inhibiting or promoting mRNA translation, stability, or degradation [32]. Therefore, changes in expression levels of miRNA can promote or inhibit tumorigenesis by altering the expression of tumor suppressors and oncogenes [33, 34]. Most recently, CSC-associated drug resistance and metastasis are being specifically targeted (reviewed in [35]) as potential treatment options. Current strategies for targeting CSCs in cancer include small molecule inhibitors, immunotherapy, microRNA mediated inhibitors, epigenetic methods as well as targeting CSC niche-microenvironmental factors and differentiation.

To capture and integrate the long list of potential factors associated with tumor progression and patient survivability, bioinformatics methods are gaining traction in the study of large gene sets, including stem cell-related gene in cancer cells [10], aimed at identifying new therapies for treating resistant cancers. Malta et. al, used an innovative one-class logistic regression (OCLR) machine-learning algorithm [36] to extract transcriptomic and epigenetic feature sets derived from pluripotent stem cells and differentiated cells, and found biological mechanisms associated with a dedifferentiated oncogenic state, called the *stemness index*. Two independent stemness indices were found, a methylated DNA stemness index (mDNAsi) highlighted the epigenetic features of the cells and a messenger RNA stemness index (mRNAsi) indicating gene expression [10]. The associations between the two stemness indices highlighted novel oncogenic pathways, somatic alterations, miRNAs and transcriptional regulatory networks, and revealed a strong negative correlation between cancer stemness and infiltrating immune cells in the tumors [10]. These advancements in bioinformatics methods, stemness index has been applied to identify key biomarkers and potential therapeutic strategies associated with cancer [30, 37–42].

Using the recently reported stem cell-related biomarkers in cells from LUAD patients [30, 43, 44]. We explored the relationship between stemness related gene and miRNA expression, and immune infiltration rates in the TMEs of LUAD patients and patient survival probability. We found that the increased expression of stemness related genes in LUAD correlated with reduced immune cell infiltration and reduced patient survivability. We also identified modulation of MDSC infiltration and hsa-miR-1247-3p expression as a key regulators in LUAD patient survival rates, potentially biomarkers for diagnoses and treatment in LUAD patients.

## Methods

### Data collection

We used 109 stem cell-related genes indicating tumor stemness from cancer transcriptomes using single-sample gene set enrichment analysis (ssGSEA) from cancer transcriptomes [9]. Gene and miRNA expression data of 537 LUAD tumor and 59 normal samples were collected from The Cancer Genome Atlas (TCGA) database [45], and two more microarray gene expression datasets were obtained from GEO database [46] (GSE ID: GSE40419 and GSE31210) and used as a validation cohort for gene differential expression test. Here GSE40419 contains 87 tumor samples and 77 normal samples, whereas GSE31210 contains 226 tumor samples and 20 normal samples. Tumor patient cancer stage (I, II, III, and IV), patient age, and LUAD specific patients survival duration (in months) were collected from cBioPortal [47].

### Differential expression test

A differential expression test of TCGA gene and miRNA expression data was performed using DeSeq2 [48] to detect differentially expressed genes and miRNA in LUAD cancer. Limma [49] was used to perform differential expression test on microarray gene expression data obtained from GEO database. Differentially expressed genes and miRNA were identified using Benjamini and Hochberg (BH) adjusted *p-value* cutoff < = 0.05 and *log2 foldchange* (LFC) >2 or LFC < –2. Here, the *foldchange* in gene and miRNA expression corresponds to the ratio between expression in cancer compared with normal samples.

### Univariate Cox-proportional hazard model-based survival analysis

Cox-proportional hazard model [50] was implemented to calculate a hazard ratio, which corresponds to the relationship between the change in expression of a gene in cancer samples and patient survival probability. A hazard rate is defined as the likelihood of experiencing a hazardous event, such as increased disease progression or death, during a defined duration time [50]. We divided patient clinical assessment and gene expression data into high and low gene expression groups and calculated a hazard rate for both groups. A hazard ratio was then calculated from the hazard rate obtained from high expression group and low expression groups. A hazard ratio greater than 1.0, with adjusted *p-value* less than 0.05, indicated a reduced probability of patient survival in the group containing higher expression of stemness genes. This step, known as survival analysis, was performed using Timer2.0 [51]. The *p-values* obtained from survival analysis were adjusted for multiple tests using Benjamini and Hochberg method. The genes with adjusted *p-value* less than 0.05 were selected as markers associated with the loss of patient survival in LUAD for downstream analysis.

### Immune infiltration test

Immune infiltration profile and its association with gene expression in LUAD patient samples were obtained from Timer2.0 (http://timer.cistrome.org/) [51], a comprehensive resource that includes data on immune and accessory cell infiltration calculated using TCGA gene expression data through various computational methods such as Timer [52], TIDE [53], MCP-counter [54], quanTIseq [55], xCell [56], CIBERSORT [57], Epic [58], and immunedeconv [59]. Computationally predicted LUAD tumor infiltration rate of a total of 32 immune and accessory cell types were examined in this study including endothelial cell, cancer associated fibroblasts, hematopoietic stem cells (HSCs), memory B cells, naïve B cell, plasma B cell, naive T cell CD4+, central memory T cell CD4+, effector memory T cell CD4+, Th1 T cell CD4+ (Th1), Th2, naive T cell CD8+, central memory T cell CD8+, effector memory T cell CD8+, Tregs, neutrophils, monocytes, macrophage M0, macrophage M1, macrophage M2, myeloid dendritic cells, plasmacytoid dendritic cells, natural killer (NK) cells, mast cells, common lymphoid progenitors, common myeloid progenitors, granulocyte-monocyte progenitor, eosinophils, T cell follicular helper, T cell gamma delta, natural killer T cell, myeloid derived suppressor cells (MDSCs). Here, immune cells with correlation greater than 0.6 or less than -0.6, and p-value < = 0.05, were used as a selection cutoff to identify highly correlated gene expression and immune cell infiltration rate pairs. Cox-proportional hazard model test and Kaplan-Meier curve calculation results were also obtained from Timer2.0 to test the relationship between high and low immune cell infiltration rates and LUAD cancer patient survival.

### Identification of miRNAs binding to the 3’ UTR region of marker genes

All miRNAs binding to 3’ UTR region of genes were identified using mirWalk [60]. Along with miRNA-gene 3’ UTR binding prediction, mirWalk looks for validated miRNA-gene binding from miRTarBase [61]. In this study, the 3p and 5p variants of miRNA that bind to the 3’ UTR region of the differentially expressed stemness-related genes were identified and selected using mirWalk and annotated as known gene targeting miRNAs in miRTarBase database. The stemness genes targeting miRNAs that exhibited a LFC greater than 2 or less than -2, with BH adjusted p-value ≤ 0.05 in both DeSeq2 and Limma differential expression tests were selected for further downstream analysis.

### Multivariate Cox-proportional hazard model-based survival analysis

Multivariate Cox-proportional hazard model-based survival analysis was performed to calculate contribution of key factors to change in patient survival rate. The data set consisted of various predictors, including patient age, cancer stages, patient survival duration after being diagnosed with LUAD, genes expression level, miRNAs expression level, and computationally predicted immune cell infiltration rates. Here, MDSC infiltration rates were collected from Tide [62]. Computationally predicted infiltration data of HSC and T-cell CD4+ Th2 (Th2) was obtained from Timer2.0 database. Multivariate Cox-proportional hazard model analysis was conducted using Survival Analysis package in R. This comprehensive approach allowed us to account for the complex interplay between the variables and their effects on cancer patient survival rate.

## Results

### Stemness genes associated with cancer patient survival

Out of total 109 stemness related genes obtained from Miranda et al., 2019 [9], four (ORC1L, KIF20A, DLGAP5, and RAB3B) genes were found to be differentially expressed in LUAD cancer using datasets TCGA, GSE40419, and GSE31210, hazard ratio > 1. Benjamini and Hochberg (BH) adjusted p-value ≤ 0.05 (Table 1). Due to their likely role in promoting cancer progression, ORC1L, KIF20A, DLGAP5, and RAB3B were selected as marker genes for downstream analysis.

**Table 1:** Differentially expressed stemness genes and regression coefficients obtained from survival analysis. Here, positive log foldchange change (LFC) corresponds to upregulated genes and negative log foldchange corresponds to downregulated genes. LUAD cancer patient data is divided into high expression and low expression groups, with low gene expression used as reference values when performing Cox-proportional hazard model analysis [51] in Timer 2.0. The hazard ratio for each gene represents the impact of the corresponding gene expression change observed in high expression sample as compared to the low expression samples within the tumor samples. Genes with log foldchange greater than 2 or less than -2 across TCGA using DeSeq2 and GSE40419 datasets and GSE31210 dataset using Limma, and with hazard ratio greater than 1 with Benjamini and Hochberg (BH) adjusted p-value less than 0.05 is selected as key genes that are associated with loss of cancer patient survival. If gene differential expression test BH adjusted p-value is not smaller than 0.05, then it is represented with ns.

	Differential expression test log foldchange				Survival Analysis		
Dataset	TCGA	TCGA	GSE40419	GSE31210	Hazard ratio	p-value	BH adjusted p-value
Method	DeSeq2	Limma	Limma	Limma			
DLGAP5	3.671	5.411	4.184	3.003	1.314	0.0	0.0
KIF20A	3.143	4.791	3.912	2.211	1.314	0.0	0.0
CENPI	2.740	3.907	2.719	ns	1.225	0.013	0.02785
ORC1L	2.741	4.081	2.564	2.530	1.263	0.002	0.006
IGF2BP1	6.589	4.805	3.485	ns	1.272	0.0	0.0
HMGA2	5.761	4.583	4.192	ns	1.14	0.001	0.00375
RAB3B	4.242	4.785	3.152	2.065	1.181	0.026	0.04875
DNMT3B	2.046	2.836	1.536	0.601	1.109	0.131	0.17863
SOHLH2	4.433	3.367	2.588	ns	1.021	0.727	0.77892
FGF2	-2.009	-3.058	-2.269	-0.828	1.217	0.11	0.165
MYCN	3.378	2.916	0.834	ns	0.94	0.275	0.32307
INHBE	4.239	3.322	0.809	ns	0.97	0.813	0.813
NMNAT2	2.735	2.252	0.771	ns	1.052	0.28	0.32307
LIN28B	7.632	5.190	3.884	ns	1.238	0.047	0.07833
ALX1	3.189	2.699	1.372	ns	1.286	0.007	0.0175

### Stemness genes and immune cell infiltration

**Fig 1:**
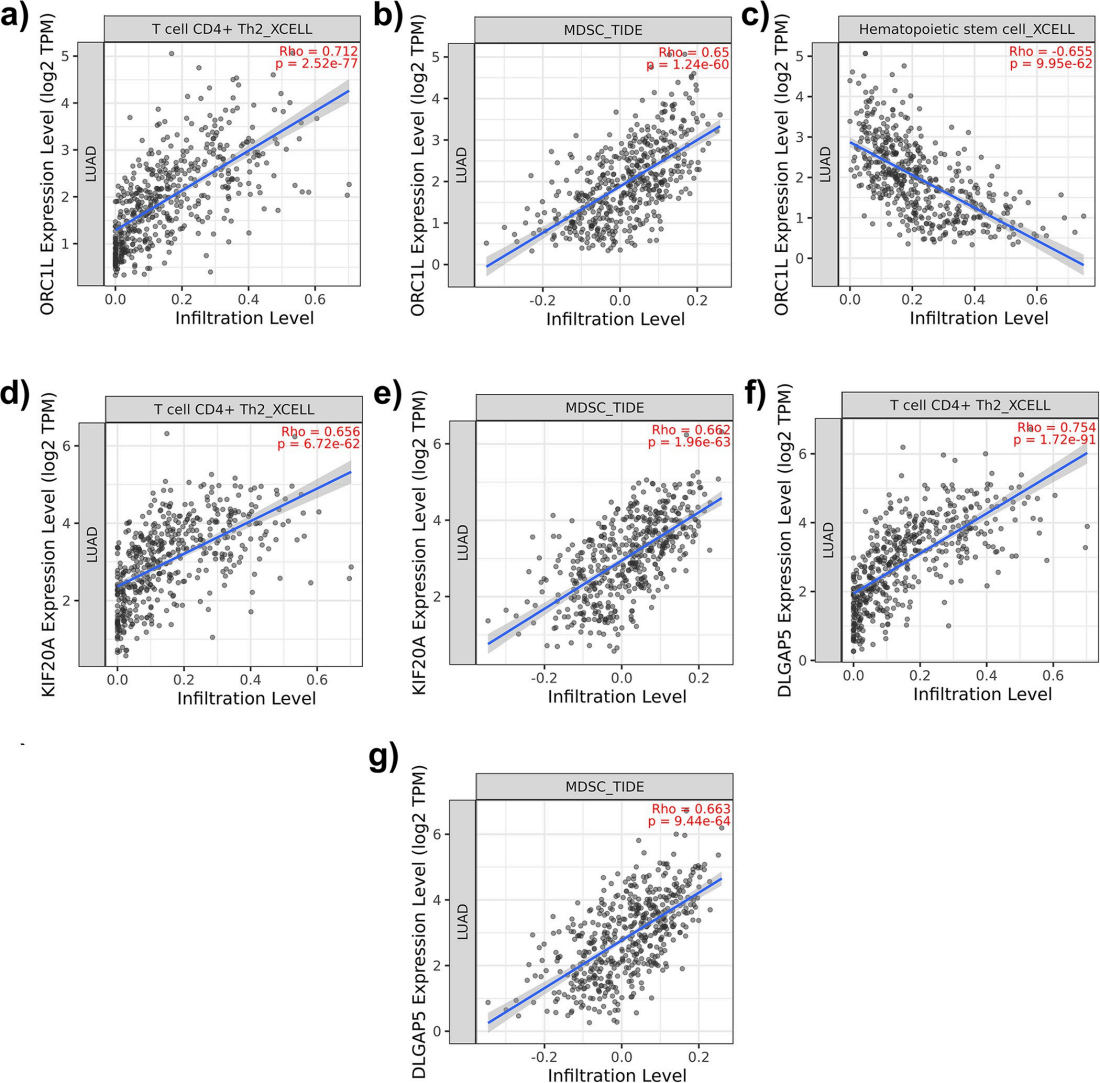
Immune cell infiltration profiles. a) correlation between T cell CD4+ Th2 (Th2) cells infiltration rate and ORC1L gene expression, b) correlation between MDSC infiltration rate and ORC1L gene expression, c) correlation between HSC infiltration rate and ORC1L gene expression, d) correlation between Th2 cells infiltration rate and KIF20A gene expression, e) correlation between MDSCs infiltration rate and KIF20A gene expression, f) correlation between Th2 cells infiltration rate and DLGAP5 gene expression, g) correlation between MDSCs infiltration rate and DLGAP5 gene expression.

Three stemness-related gene (ORC1L, KIF20A, DLGAP5) positively correlated (> 0.6) with the infiltration rate of Th2 and MDSC. ORC1L negatively correlated with hematopoietic stem cell (HSC) infiltration rate (< -0.6) (Fig 1). Cox-proportional hazard Model-based survival analysis coefficients obtained from Timer2.0 indicated a higher infiltration rate of both Th2 and MDSCs in three genes (ORC1L, KIF20A, and DLCAP5) corresponding to a lower patient survival rate. RAB3B did not exhibit a correlation with any immune and accessory cell infiltration rate within the correlation cutoff range. Moreover, higher infiltration rate of HSCs corresponded to higher patient survival rate (Table 2).

**Table 2:** Hazard ratio and p-value of Cox-proportional hazard model test for Th2, MDSC, and HSC cell immune infiltration rate with patient survival report. Here, LUAD cancer patient data is divided into high and low infiltration groups with low immune cell infiltration is used as reference value when performing Cox-proportional hazard model analysis in Timer2.0 [51].

Factor name	Reference value	Factor value	Hazard ratio	pvalue	BH adjusted p-value
Th2	Low infiltration	High infiltration	3.804	0.006	0.009
MDSC	Low infiltration	High infiltration	62.036	0	0
HSC	Low infiltration	High infiltration	0.167	0.046	0.046

### miRNAs regulating stemness genes

From cross-referencing the mirWalk and miRTarBase database [61], total five miRNAs (hsa-mir-197-3p, hsa-mir-1247-3p, hsa-mir-122-5p, hsa-mir-215-5p, hsa-mir-192-5p) were differentially expressed in TCGA dataset DeSeq2 results, all targeting 3’ UTR region of RAB3B (Table 3). No miRNAs targeting ORC1L, KIF20A, and DLGAP5 3’ UTR regions were differentially expressed in TCGA dataset in DeSeq2 analysis results. Differential expressions of these 5 miRNAs in the TCGA dataset were also conducted using Limma to remove potential false positives. As RAB3B is upregulated in LUAD, downregulated miRNAs with Limma LFC less than -2, and BH adjusted p-value ≤0.05, were selected for downstream analysis. Out of these 5 miRNAs, only one, hsa-mir-1247-3p, exhibited the Limma LFC and BH adjusted p-value within the cutoff criteria (Table 3).

**Table 3:** Log2 foldchange (LFC) and Benjamini/Hochberg (BH) adjusted p-value obtained from DeSeq2 and Limma for 5 miRNAs. Here, miRNAs with both DeSeq2 and Limma log foldchange greater than 2 or less than -2, and BH adjusted p-value ≤0.05 were selected for downstream analysis.

miRNAs		DeSeq2		Limma	
	RAB3B binding miRNA variant	LFC	BH adjusted p-value	LFC	BH adjusted p-value
hsa-mir-1247	3p	-2.324	3.51E-19	-3.151	1.63E-11
hsa-mir-192	5p	3.162	1.62E-21	2.715	4.19E-08
hsa-mir-122	3p	1.806	9.95E-08	2.007	0.245932
hsa-mir-215	5p	2.135	2.51E-09	0.655	0.245932
hsa-mir-197	3p	-2.965	1.58E-79	0.130	0.628424

### Key markers of LUAD

Multivariate Cox-proportional hazard model analysis was first conducted using hsa-mir-1247-3p, KIF20A, RAB3B, ORC1L, and DLGAP5 expression, MDSC, Th2, and HSC infiltration, LUAD cancer patient age and cancer stage as covariates to identify key markers promoting loss of patient survival rate. Results (Table 4) indicate that although hsa-mir-1247-3p was downregulated in LUAD, increases in hsa-mir-1247 expression within the cancer patient samples lead to loss of survival rate (Table 4). Furthermore, the increase in infiltration of MDSC is also shown to be associated with LUAD loss of survival rate (Table 4). No significant correlation (Pearson correlation score = -0.05) between MDSC, and hsa-mir-1247-3p was observed. Furthermore, as expected, cancer stage II, III, and IV and increased age are significantly associated with a decrease in cancer patient survival rate (Table 4). These results suggest that modulation of hsa-mir-1247-3p expression and MDSC infiltration significantly contributed to the loss of patient survival in LUAD, but likely use distinctly different mechanisms.

**Table 4:** Multivariate Cox-proportional hazard model analysis results highlighting key markers associated with increase in patient mortality rate. Here, LUAD patient tumor samples are divided into low and high infiltration groups for immune cell infiltration, low and high expression groups for gene and miRNA expression, and low and high age groups for LUAD cancer patient age. Reference values used for multivariate Cox-proportional hazard model analysis for each factor are shown in the table.

Factor name	Reference value	Factor value	HR	p-value
Cancer stage	STAGE I	STAGE III	3.17373	6.30E-08
Cancer stage	STAGE I	STAGE IV	3.627736	2.46E-05
Cancer stage	STAGE I	STAGE II	1.941041	0.001473
hsa-mir-1247-3p	High expression	Low expression	0.667653	0.02279
MDSC	High infiltration	Low infiltration	0.655898	0.028825
Age	High	Low	0.740296	0.071889
Th2	High infiltration	Low infiltration	0.829268	0.361647
KIF20A	High expression	Low expression	0.807105	0.428903
RAB3B	High expression	Low expression	0.892945	0.536809
HSC	High infiltration	Low infiltration	1.048088	0.827427
ORC1L	High expression	Low expression	0.964545	0.881234
DLGAP5	High expression	Low expression	0.981126	0.944834

### Role of hsa-mir-1247-3p target genes in LUAD

**Fig 2:**
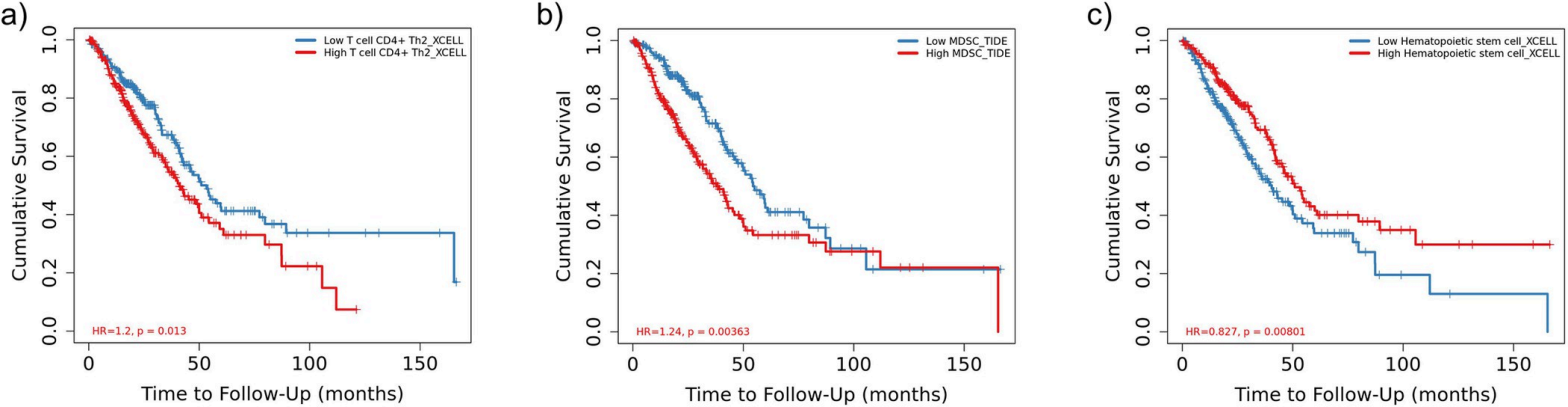
Immune cell infiltration profiles. a) correlation between T-cell CD4+ Th2 (Th2) cells infiltration rate and HJURP gene expression, b) correlation between MDSC infiltration rate and HJURP gene expression, c) correlation between HSC infiltration rate and HJURP gene expression.

A total of 53 genes were identified as common targets of has-mir-1247-3p in both the mirWalk and mirTarBase databases (S1 Table). Differential expression analysis of the TCGA, GSE40419, and GSE31210 datasets of these 53 genes, utilizing both DeSeq2 and Limma methods, showed that HJURP and RAB3B consistently exhibited a LFC greater than 2.0 across all datasets (S2 Table). Additionally, SLC24A4 showed an LFC of less than -2.0 across all three datasets when analyzed with the Limma, and an LFC of -1.98158 when assessed using the DeSeq2 (S2 Table). These results indicate that in addition to RAB3B, two other hsa-mir-1247-3p target genes HJURP and SLC24A4, are also significantly differentially expressed across all examined datasets and methodological approaches. Like expression trends of ORC1L, HJURP exhibited significant positive correlation with Th2 and MDSC, and a significant negative correlation with HSC (Fig 2). However, SLC24A4 did not exhibit a correlation greater than 0.6 or less than -0.6 with any immune and accessory cell infiltration rates. Additionally, hsa-mir-1247-3p, binding to the 3’ UTR region of the STAT5A gene, has also been suggested to play a role in reducing the progression of LUAD [63]. Here, univariate Cox-proportional hazard model analysis results obtained from Timer2.0 database indicates that change expression of HJURP and SALC24A4 is significantly associated with loss of LUAD cancer patient survival rate, whereas STAT5A exhibited a weaker association trend with patient survival rate (Table 5). A multivariate Cox-proportional hazard model analysis incorporating factors including hsa-mir-1247-3p, RAB3B, HJURP, SLC24A4, and STAT5A expression, cancer stage, patient age, and MDSC, Th2, and HSC infiltration rates, was conducted to assess the specific influence of HJURP, SLC24A4, and STAT5A on patient survival. Genes HJURP, STAT5A, and SLC24A4, like RAB3B, also failed to demonstrate a significant trend of association with the loss of patient survival rate (Table 6).

**Table 5:** Hazard ratio and p-value of Cox-proportional hazard model test for HJURP, SLC24A4, and STAT5A expression with patient survival report. Here, LUAD cancer patient data is divided into high and low and high expression groups, where low expression is used as reference value when performing Cox-proportional hazard model analysis in Timer2.0 [51].

Factor name	Reference value	Factor value	HR	p-value
HJURP	Low expression	High expression	1.29	0
SLC24A4	Low expression	High expression	0.399	0.013
STAT5A	Low expression	High expression	0.868	0.14

**Table 6:** Multivariate Cox-proportional hazard model analysis results of differentially expressed genes targeted by hsa-mir-1247-3p. Here, LUAD patient tumor samples are divided into low and high infiltration groups for immune cell infiltration, low and high expression groups for gene and miRNA expression, and low and high age groups for LUAD cancer patient age. Reference values used for multivariate Cox-proportional hazard model analysis for each factor are shown in the table.

Factor name	Reference value	Factor value	HR	p-value
Cancer stage	STAGE I	STAGE III	3.060216	1.49E-07
Cancer stage	STAGE I	STAGE IV	3.666859	2.08E-05
Cancer stage	STAGE I	STAGE II	1.938426	0.001264
hsa-mir-1247-3p	High expression	Low expression	0.675477	0.02661
MDSC	High infiltration	Low infiltration	0.681396	0.084532
Age	High	Low	0.763052	0.102087
HJURP	High expression	Low expression	0.709094	0.121995
Th2	High infiltration	Low infiltration	0.858448	0.456893
SLC24A4	High expression	Low expression	1.117642	0.579472
STAT5A	High expression	Low expression	0.926444	0.682518
RAB3B	High expression	Low expression	0.934566	0.712562
HSC	High infiltration	Low infiltration	1.039916	0.852716

## Discussion and conclusions

Using predefined stemness-related genes and cross-referencing these against the LUAD patients, we found 15 differentially expressed stemness-related genes in LUAD. Of these, four genes (ORC1L, KIF20A, DLGAP5, and RAB3B) negatively correlated with the probability of patient survival (Table 1 and Fig 1). We then examined the immune cell infiltration in LUAD cancer patients and found a decreased infiltration rate of HSCs and increased infiltration of MDSC and Th2 cells correlating with decreased survival probability correlating with three genes (ORC1L, KIF20A, and DLGAP5—Fig 1). Lastly, the examination of stemness-related genes and immune cell infiltration rates in LAUD patients linked the increase in expression of ORC1L with the negatively correlated infiltration rate of HSC (Fig 2C) but did not suggest a strong correlation between Th2 infiltration or other differentially expressed stemness-related genes.

ORC1L is a highly conserved six subunit protein, which plays an important role in regulating initiation of DNA replication process during cell division [64] and organ development [65]. Recently, aberrations in ORC1L have been implicated in breast cancer [66], cervical cancer [67], as well as LUAD cancer progression [68]. Investigating the role of ORC1L more closely, we found that aberrations in ORC1L gene expression have been previously linked with LUAD tumor growth [68, 69] through changes in immune cell infiltration [68]. Han et al., [68] postulated that ORC1L promotes LUAD tumor progression and aberration of immune infiltration by deregulating WNT signaling pathway. Alterations of WNT signaling have been implicated in modulation of immune infiltration in cancer, thereby promoting tumor progression [70]. Therefore, the relationship between the upregulation of ORC1L and changes in immune infiltration and loss LUAD patient survival is likely driven by changes in the WNT signaling pathway.

As expected, our results also agreed with previous studies [22, 25–27, 30] showing that an increase in infiltration of MDSC and Th2 decreased the survival probability of cancer patients in LUAD (Fig 2). Uniquely, we found an increase in expression of ORC1L, KIF20A, and DLGAP5 stemness-related genes also positively correlated with MDSC and Th2 infiltration rates in LUAD cancer (Fig 1). Myeloid-derived suppressor cells (MDSCs) comprise a varied collection of undeveloped myeloid cells and play a crucial role in negatively modulating the immune response [71]. This regulation, combined with immune infiltration rates of T-helper Th2 cells, contributes to the advancement of tumors [71], the formation of pre-metastatic niches [72], and a decrease in the effectiveness of immunotherapy [71]. The underlying processes that achieve these functions are intricate and multifaceted, encompassing both immunosuppressive activities (such as the restraint of cytotoxic T-cells [73]) and non-immunological roles like facilitating stemness genes [74] and encouraging angiogenesis by secreting various growth factors such as vascular endothelial growth factor (VEGF), basic fibroblast growth factor (bFGF), prokineticin-2 (Bv8), and platelet-derived growth factor (PDGF) [75]. The MDSC activation and expansion by stemness-related genes [26] has also been shown to negatively regulate the host’s immune response by promoting release of cytokines and chemokines to protect cancer cells in many cancers [76]. Similarly, upregulation of KIF20A stemness-related gene is known to positively correlate with Th2, Treg cells and macrophage infiltration rates, and negatively correlate with Th17, mast cells and natural killer cells infiltration in renal cell carcinoma [77]. This meta-analysis confirmed the similar role for KIF20A in LUAD.

Looking more closely at the two mitosis related gene, DLGAP5, it has also been shown to be important in cancer prognosis [78, 79], but the mechanism associated with its impact on cancer progression remains unclear. This meta-analysis found the expression of DLGAP5 is positively correlated with Th2 and MDSC infiltration rate, as well as loss of patient survival probability, highlighting its role in promoting cancer progression. DLGAP5 is a microtubule associated protein, which maintains progression of normal cell division by regulating chromosomal rearrangement and gene stability [80]. Looking at current literature, the overexpression of DLGAP5 has been shown to increase cancer cell migration and invasion in gastric cancer [81].

Searching the miRNA database for potential miRNAs correlating positively with stemness-related genes in LUAD cancer, we found one miRNA (has-mir-1247-3p) targeting RAB3B, HJURP, SLC24A4, and STAT5A, all differentially expressed in LUAD. Previous reports on hsa-mir-1247-3p found it to be a key biomarker in pancreatic cancer [82], breast cancer [83], liver cancer [84], bone cancer [85], and neuroblastoma [86]. Moreover, deregulation of hsa-mir-1247-3p has been shown to lead to increased cancer progression [63] suggesting that it may play a role in mitigating cancer progression in LUAD cancers [63] (correlation coefficient -0.314, p-value 0.001 with STATA5A gene expression).

Despite the downregulation of hsa-mir-1247-3p in LUAD, the multivariate Cox-proportional hazard model analysis suggested that an increase in expression of hsa-mir-1247-3p within LUAD patients had a significant association with loss of patient survivability (Tables 4 and 5). Upon further investigation further, it seems that miRNAs are known to regulate oncogenes as well as tumor suppressors, and the balance between the two mediates direction of impact of tumor progression [87]. Results obtained from this study, and Lin et al., [63], indicate that hsa-mir-1247-3p targets LUAD differentially expressed oncogenes (RAB3B and HJURP), as well as differentially expressed tumor suppressors (SLC24A4 and STAT5A –Table 5). While the increase in expression of oncogenes RAB3B and HJURP and loss of expression of tumor suppressor SLC24A4 is associated with diminished cancer patient survivability, the downregulation of tumor suppressor STAT5A has been shown to promote tumor growth [63]. Moreover, hsa-mir-1247-3p modulation mediates the association between the change in expression of these genes and patient survival rate, acting as a key marker and central node in the regulatory network associated with tumor progression.

While traditionally considered as gene-regulatory elements, miRNAs—like hsa-mir-1247-3p - may also influence more complex signaling pathways within the tumor microenvironment. These analyses did not indicate a significant correlation between MDSC infiltration scores and hsa-mir-1247-3p expression, suggesting distinct regulatory mechanisms leading to their shared involvement in regulating patient survival rate.

## Supporting information

S1 Table List of genes targeted at 3’ UTR region by hsa-mir-1247-3p obtained from mirWalk database [1]. Here, each gene-miRNA pair is a validated interaction with mirTarBase [2] ID.
(DOCX)

S2 Table Differential expression results of genes targeted by hsa-mir-1247-3p at 3’ UTR regions. Here, log2 foldchange (LFC) and Benjamini and Hochberg (BH) adjusted p-values were obtained from DeSeq2 [1] and Limma [2] applied on TCGA [3] and GEO [4] datasets.
(DOCX)

## Abbreviations

CSC: cancer stem cell
HSC: hematopoietic stem cell
LFC: Log2 Fold Change
LUAD: Lung adenocarcinoma
mRNA: messenger RNA
miRNA: microRNA
MDSC: myeloid-derived suppressor cells
TME: tumor microenvironment
Th2: Th2 T cell CD4+
TCGA: The Cancer Genome Atlas
