# Supplementary material for: Stemness genes and miR-1247-3p expression associate with clinicopathological parameters and prognosis in lung adenocarcinoma
Source: PLoS One. 2023 Nov 10;18(11):e0294171. doi: 10.1371/journal.pone.0294171 (PMC10637681; doi:10.1371/journal.pone.0294171)
Supplement: S1 Table — Here, each gene-miRNA pair is a validated interaction with mirTarBase [2] ID. (DOCX) [file pone.0294171.s001.docx]

**Supplementary Material**

**S1 Table.** **List of genes targeted at 3’ UTR region by hsa-mir-1247-3p obtained from mirWalk database** [1]. Here, each gene-miRNA pair is a validated interaction with mirTarBase [2] ID.

| **Mirna ID** | **Gene symbol** | **Binding position** | **mirTarBase ID** |
| --- | --- | --- | --- |
| hsa-miR-1247-3p | TMEM106B | 3UTR | MIRT679827 |
| hsa-miR-1247-3p | PBX1 | 3UTR | MIRT736387 |
| hsa-miR-1247-3p | DUSP19 | 3UTR | MIRT457322 |
| hsa-miR-1247-3p | ORAI2 | 3UTR | MIRT701215 |
| hsa-miR-1247-3p | TP53 | 3UTR | MIRT790057 |
| hsa-miR-1247-3p | MDM4 | 3UTR | MIRT473408 |
| hsa-miR-1247-3p | HJURP | 3UTR | MIRT691139 |
| hsa-miR-1247-3p | CCSER2 | 3UTR | MIRT479671 |
| hsa-miR-1247-3p | EVI5 | 3UTR | MIRT736378 |
| hsa-miR-1247-3p | HMGB1 | 3UTR | MIRT678239 |
| hsa-miR-1247-3p | S100A16 | 3UTR | MIRT761563 |
| hsa-miR-1247-3p | CENPI | 3UTR | MIRT761523 |
| hsa-miR-1247-3p | LYRM4 | 3UTR | MIRT458160 |
| hsa-miR-1247-3p | ZC3H8 | 3UTR | MIRT662136 |
| hsa-miR-1247-3p | SPPL2A | 3UTR | MIRT520971 |
| hsa-miR-1247-3p | GTPBP10 | 3UTR | MIRT761538 |
| hsa-miR-1247-3p | ZFP82 | 3UTR | MIRT631356 |
| hsa-miR-1247-3p | SLC30A7 | 3UTR | MIRT761571 |
| hsa-miR-1247-3p | DTX3L | 3UTR | MIRT460020 |
| hsa-miR-1247-3p | CCDC127 | 3UTR | MIRT736374 |
| hsa-miR-1247-3p | ZNF585B | 3UTR | MIRT452065 |
| hsa-miR-1247-3p | SLC24A4 | 3UTR | MIRT691780 |
| hsa-miR-1247-3p | HSD17B13 | 3UTR | MIRT761541 |
| hsa-miR-1247-3p | BLOC1S3 | 3UTR | MIRT118004 |
| hsa-miR-1247-3p | AVL9 | 3UTR | MIRT481160 |
| hsa-miR-1247-3p | THAP6 | 3UTR | MIRT761576 |
| hsa-miR-1247-3p | ZMYM1 | 3UTR | MIRT676851 |
| hsa-miR-1247-3p | SNX22 | 3UTR | MIRT458465 |
| hsa-miR-1247-3p | SLC7A11 | 3UTR | MIRT677473 |
| hsa-miR-1247-3p | DNTTIP2 | 3UTR | MIRT761528 |
| hsa-miR-1247-3p | PMPCA | 3UTR | MIRT550734 |
| hsa-miR-1247-3p | MOB4 | 3UTR | MIRT671010 |
| hsa-miR-1247-3p | PLCXD1 | 3UTR | MIRT761557 |
| hsa-miR-1247-3p | TIGAR | 3UTR | MIRT761577 |
| hsa-miR-1247-3p | ZNF70 | 3UTR | MIRT463022 |
| hsa-miR-1247-3p | ZCCHC4 | 3UTR | MIRT736404 |
| hsa-miR-1247-3p | CPM | 3UTR | MIRT761526 |
| hsa-miR-1247-3p | POU3F1 | 3UTR | MIRT613920 |
| hsa-miR-1247-3p | RAB3B | 3UTR | MIRT676855 |
| hsa-miR-1247-3p | SPI1 | 3UTR | MIRT625720 |
| hsa-miR-1247-3p | ACOX1 | 3UTR | MIRT559740 |
| hsa-miR-1247-3p | STK17B | 3UTR | MIRT460978 |
| hsa-miR-1247-3p | ATXN3 | 3UTR | MIRT512229 |
| hsa-miR-1247-3p | AKR1D1 | 3UTR | MIRT624770 |
| hsa-miR-1247-3p | CTSV | 3UTR | MIRT461421 |
| hsa-miR-1247-3p | SRGAP1 | 3UTR | MIRT467127 |
| hsa-miR-1247-3p | ATP6V1B1 | 3UTR | MIRT568911 |
| hsa-miR-1247-3p | LDLR | 3UTR | MIRT633264 |
| hsa-miR-1247-3p | CRX | 3UTR | MIRT736376 |
| hsa-miR-1247-3p | ZNF805 | 3UTR | MIRT761595 |
| hsa-miR-1247-3p | CRCP | 3UTR | MIRT634753 |
| hsa-miR-1247-3p | ZNF554 | 3UTR | MIRT675205 |
| hsa-miR-1247-3p | DNAL1 | 3UTR | MIRT478006 |

**References**

1. Sticht C, De La Torre C, Parveen A, Gretz N. Mirwalk: An online resource for prediction of microrna binding sites. PLoS One. 2018;13: e0206239. doi:10.1371/journal.pone.0206239

2. Huang HY, Lin YCD, Li J, Huang KY, Shrestha S, Hong HC, et al. MiRTarBase 2020: Updates to the experimentally validated microRNA-target interaction database. Nucleic Acids Res. 2020;48: D148–D154. doi:10.1093/nar/gkz896
