# Supplementary material for: Stemness genes and miR-1247-3p expression associate with clinicopathological parameters and prognosis in lung adenocarcinoma
Source: PLoS One. 2023 Nov 10;18(11):e0294171. doi: 10.1371/journal.pone.0294171 (PMC10637681; doi:10.1371/journal.pone.0294171)
Supplement: S2 Table — Here, log2 foldchange (LFC) and Benjamini and Hochberg (BH) adjusted p-values were obtained from DeSeq2 [1] and Limma [2] applied on TCGA [3] and GEO [4] datasets. (DOCX) [file pone.0294171.s002.docx]

**Supplementary Material**

**S2 Table.** **Differential expression results of genes targeted by hsa-mir-1247-3p at 3’ UTR regions.** Here, log2 foldchange (LFC) and Benjamini and Hochberg (BH) adjusted p-values were obtained from DeSeq2 [1] and Limma [2] applied on TCGA[3] and GEO[4] datasets.

| **Dataset** | **TCGA** | | | | **GSE40419** | | **GSE31210** | |
| --- | --- | --- | --- | --- | --- | --- | --- | --- |
| **Method** | **DeSeq2** | | **Limma** | | **Limma** | | **Limma** | |
|  | **LFC** | **BH adjusted p-value** | **LFC** | **BH adjusted p-value** | **LFC** | **BH adjusted p-value** | **LFC** | **BH adjusted p-value** |
| **HJURP** | 3.852076 | 3.2E-105 | 3.905457 | 5.27E-61 | 4.254974 | 2.6E-31 | 2.262547 | 2.18E-07 |
| **CENPI** | 2.744335 | 9.97E-69 | 2.708458 | 8.50E-42 | 2.719357 | 2.89E-20 | 0.674653 | 0.207698 |
| **SNX22** | -2.24337 | 8.95E-49 | -2.16948 | 5.50E-32 | 0.440408 | 0.000181 | -0.86776 | 0.017666 |
| **AVL9** | 1.098825 | 3.26E-38 | 1.356479 | 1.73E-29 | 1.366566 | 3.94E-18 | 0.032749 | 0.964102 |
| **ZC3H8** | 0.821771 | 8.88E-26 | 1.166931 | 2.37E-25 | 1.329108 | 1.19E-23 | 0.589307 | 9.17E-05 |
| **SLC24A4** | -1.98158 | 7.69E-29 | -2.15919 | 2.37E-25 | -2.04999 | 3.49E-23 | -2.86014 | 1.44E-08 |
| **LDLR** | -2.12195 | 3.97E-58 | -1.71552 | 2.12E-24 | -1.70458 | 4.58E-15 | -1.10555 | 0.003086 |
| **SLC7A11** | 3.073835 | 1.87E-36 | 2.686261 | 4.97E-20 | 3.154275 | 1.68E-18 | 1.205486 | 0.01074 |
| **RAB3B** | 4.242609 | 3.16E-43 | 3.316887 | 1.15E-19 | 3.152845 | 2.62E-14 | 2.065086 | 8.42E-05 |
| **TMEM106B** | 0.88544 | 1E-16 | 1.09891 | 6.77E-18 | 0.698497 | 3.86E-05 | 1.854801 | 3.23E-14 |
| **ZMYM1** | 0.672395 | 1.87E-16 | 0.963426 | 1.73E-17 | 0.826195 | 4.43E-07 | 0.422951 | 0.029073 |
| **SPI1** | -1.58377 | 1.81E-29 | -1.46026 | 1.94E-14 | -1.67022 | 1.3E-14 | 0.00389 | 0.994909 |
| **GTPBP10** | 0.472117 | 4.59E-10 | 0.776281 | 6.59E-14 | 0.599768 | 1.4E-07 | -1.466 | 0.019514 |
| **CTSV** | 2.259804 | 2.57E-22 | 1.957593 | 1.16E-13 | #N/A | #N/A | 0.939582 | 0.042415 |
| **SLC30A7** | 0.499429 | 7.25E-09 | 0.770074 | 6.23E-13 | 0.538635 | 8.82E-06 | 0.207454 | 0.153462 |
| **ZCCHC4** | 0.429218 | 3.69E-10 | 0.748122 | 6.23E-13 | 0.243992 | 0.00156 | 0.205289 | 0.186879 |
| **HSD17B13** | -1.4691 | 1.42E-06 | -2.13372 | 9.89E-13 | -1.59466 | 1.48E-05 | #N/A | #N/A |
| **ZNF70** | 0.480695 | 5.03E-09 | 0.824265 | 1.18E-12 | 0.435984 | 0.002031 | 0.017652 | 0.936787 |
| **CRCP** | 0.399538 | 5.18E-10 | 0.714505 | 1.34E-11 | 0.450351 | 5.2E-09 | 0.416493 | 0.009067 |
| **CCDC127** | 0.456398 | 1.44E-08 | 0.734956 | 1.71E-10 | 0.50385 | 2.28E-07 | 0.824754 | 2.34E-06 |
| **PMPCA** | 0.420262 | 1.65E-08 | 0.714321 | 3.97E-09 | 0.356805 | 4.46E-05 | 0.3913 | 0.00242 |
| **S100A16** | 1.23815 | 1.06E-14 | 1.166778 | 1.56E-08 | 0.967727 | 3.09E-08 | 0.70464 | 0.005386 |
| **THAP6** | 0.218109 | 0.001829 | 0.53881 | 2.44E-08 | 0.560088 | 3.7E-07 | 0.380402 | 0.003878 |
| **MDM4** | 0.478123 | 3.85E-05 | 0.7101 | 4.42E-08 | 0.306912 | 0.002434 | 0.101399 | 0.758991 |
| **ORAI2** | 0.445262 | 9.12E-06 | 0.696861 | 5.84E-07 | 0.273101 | 0.030545 | 0.049471 | 0.802463 |
| **DTX3L** | 0.291768 | 0.000358 | 0.57561 | 8.47E-07 | 0.426368 | 0.000104 | 0.417453 | 0.012707 |
| **TP53** | 0.528623 | 1.16E-06 | 0.710362 | 1.12E-06 | 0.80869 | 5.75E-09 | 0.862159 | 5.2E-05 |
| **LYRM4** | 0.315927 | 0.002399 | 0.596792 | 2.28E-06 | 0.09486 | 0.355914 | 0.262962 | 0.144597 |
| **AKR1D1** | 2.408048 | 1.1E-12 | 1.286543 | 1.94E-05 | 0.655824 | 0.112664 | 0.133417 | 0.84327 |
| **DNTTIP2** | 0.078411 | 0.339129 | 0.413009 | 0.0001 | 0.138115 | 0.20941 | -0.1653 | 0.259261 |
| **CPM** | -0.6187 | 0.000512 | -0.78447 | 0.000162 | -1.03446 | 0.000117 | -0.84454 | 0.092732 |
| **ZNF585B** | 0.235038 | 0.016562 | 0.48085 | 0.000162 | 0.160386 | 0.364974 | -0.0866 | 0.697104 |
| **ACOX1** | 0.100567 | 0.209876 | 0.3923 | 0.000193 | 0.184445 | 0.01887 | -0.35935 | 0.550238 |
| **DUSP19** | -0.76608 | 3.35E-11 | -0.50978 | 0.000212 | -0.77964 | 1.68E-05 | -0.88738 | 0.000706 |
| **PBX1** | -0.68168 | 1.42E-06 | -0.62947 | 0.000342 | -1.46615 | 1.09E-17 | -1.00116 | 0.002579 |
| **POU3F1** | 0.85551 | 4.35E-05 | 0.768486 | 0.000453 | 0.438925 | 0.190277 | 0.064559 | 0.8989 |
| **ZNF805** | 0.174936 | 0.11721 | 0.387198 | 0.000711 | 0.127943 | 0.09803 | -0.08353 | 0.649668 |
| **BLOC1S3** | 0.07203 | 0.430206 | 0.394501 | 0.001346 | 0.236942 | 0.016321 | 0.342252 | 0.01916 |
| **EVI5** | -0.55453 | 3.43E-10 | -0.29851 | 0.003993 | -0.5477 | 1.03E-06 | 0.045426 | 0.845545 |
| **SRGAP1** | 0.329459 | 0.020135 | 0.444158 | 0.005294 | -0.51537 | 0.003454 | -0.89831 | 0.021421 |
| **ZFP82** | 0.264368 | 0.041415 | 0.394416 | 0.010003 | 0.086938 | 0.608432 | 0.106893 | 0.699316 |
| **DNAL1** | -0.62377 | 7.49E-16 | -0.26479 | 0.012975 | -0.35879 | 0.005429 | 0.085277 | 0.728068 |
| **ATP6V1B1** | 0.659028 | 0.017796 | 0.494585 | 0.055935 | -1.03616 | 0.002218 | -0.99112 | 0.036977 |
| **CRX** | 0.222949 | 0.510211 | 0.238503 | 0.112992 | 0.094132 | 0.496752 | 0.378008 | 0.455369 |
| **ZNF554** | -0.11976 | 0.15796 | 0.200598 | 0.086436 | 0.048903 | 0.717643 | 0.409442 | 0.436122 |
| **TIGAR** | -0.46547 | 8.62E-07 | -0.18385 | 0.120029 | #N/A | #N/A | #N/A | #N/A |
| **SPPL2A** | -0.19318 | 0.007522 | 0.115473 | 0.255097 | 0.175867 | 0.101035 | 0.391454 | 0.015313 |
| **MOB4** | -0.23588 | 0.004574 | 0.11809 | 0.255097 | #N/A | #N/A | -0.3519 | 0.010121 |
| **HMGB1** | -0.23702 | 0.001414 | 0.085797 | 0.390494 | -0.22531 | 0.017607 | 0.025955 | 0.889045 |
| **STK17B** | -0.36712 | 0.00036 | -0.10135 | 0.40029 | -0.65982 | 3.08E-06 | 0.470229 | 0.092735 |
| **CCSER2** | -0.31154 | 0.000796 | -0.03879 | 0.738212 | #N/A | #N/A | 0.395316 | 0.340647 |
| **ATXN3** | -0.33379 | 8.85E-06 | -0.0228 | 0.817401 | -0.65473 | 7.72E-10 | -0.26711 | 0.130368 |
| **PLCXD1** | 0.30464 | 0.095809 | NA | NA | NA | NA | 0.357461 | 0.294047 |

**References**

1. Love MI, Huber W, Anders S. Moderated estimation of fold change and dispersion for RNA-seq data with DESeq2. Genome Biol. 2014;15: R106. doi:10.1186/s13059-014-0550-8

2. Ritchie ME, Phipson B, Wu D, Hu Y, Law CW, Shi W, et al. Limma powers differential expression analyses for RNA-sequencing and microarray studies. Nucleic Acids Res. 2015;43: e47. doi:10.1093/nar/gkv007

3. Weinstein JN, Collisson EA, Mills GB, Shaw KRM, Ozenberger BA, Ellrott K, et al. The cancer genome atlas pan-cancer analysis project. Nat Genet. 2013;45: 1113–1120. doi:10.1038/ng.2764

4. Barrett T, Wilhite SE, Ledoux P, Evangelista C, Kim IF, Tomashevsky M, et al. NCBI GEO: Archive for functional genomics data sets - Update. Nucleic Acids Res. 2013;41: D991–D995. doi:10.1093/nar/gks1193
